# Over-Expression of GmGIa-Regulated Soybean *miR172a* Confers Early Flowering in Transgenic *Arabidopsis thaliana*

**DOI:** 10.3390/ijms17050645

**Published:** 2016-04-29

**Authors:** Tao Wang, Ming-Yang Sun, Xue-Song Wang, Wen-Bin Li, Yong-Guang Li

**Affiliations:** Key Laboratory of Soybean Biology in Chinese Education Ministry (Northeastern Key Laboratory of Soybean Biology and Genetics & Breeding in Chinese Ministry of Agriculture), Northeast Agricultural University, Harbin 150030, China; wangtao6559@126.com (T.W.); sunmingyangphy@163.com (M.-Y.S.); cedarwxs@163.com (X.-S.W.)

**Keywords:** *Arabidopsis thaliana*, flowering, *gma-miR172a*, soybean (*Glycine max* L. Merr.)

## Abstract

Flowering is a pivotal event in the life cycle of plants. *miR172* has been widely confirmed to play critical roles in flowering time control by regulating its target gene expression in Arabidopsis. However, the role of its counterpart in soybean remains largely unclear. In the present study, we found that the *gma-miR172a* was regulated by a *GIGANTEA* ortholog, *GmGIa*, in soybean through miRNA metabolism. The expression analysis revealed that *gma-miR172a* has a pattern of diurnal rhythm expression and its abundance increased rapidly as plants grew until the initiation of flowering phase in soybean. One target gene of *gma-miR172a*, *Glyma03g33470*, was predicted and verified using a modified RLM 5′-RACE (RNA ligase-mediated rapid amplification of 5′ cDNA ends) assay. Overexpression of *gma-miR172a* exhibited an early flowering phenotype and the expression of *FT*, *AP1* and *LFY* were simultaneously increased in *gma-miR172a*-transgenic Arabidopsis plants, suggesting that the early flowering phenotype was associated with up-regulation of these genes. The overexpression of the *gma-miR172a*-resistant version of *Glyma03g33470* weakened early flowering phenotype in the *toe1* mutant of Arabidopsis. Taken together, our results suggested that *gma-miR172a* played an important role in *GmGIa*-mediated flowering by repressing *Glyma03g33470*, which in turn increased the expression of *FT*, *AP1* and *LFY* to promote flowering in soybean.

## 1. Introduction

The timing of the switch from vegetative to reproductive growth is critical for the success of plant reproduction. Flowering time is regulated by coordinated interactions between various endogenous signals and environmental cues [1,2,3]. Genetic and molecular analyses had revealed that four major pathways regulate this transition: the endogenous factors include autonomous and the gibberellin pathways, while the photoperiod and vernalization pathways respond to environmental cues [4,5,6,7]. These pathways are generally controlled by multiple genes and are influenced by the environment.

Recently, microRNAs (miRNAs), a class of small non-coding RNA molecules ranging from 18 to 24 nucleotides in length, have been identified as the key regulators of gene expression in both plants and animals [3]. Some non-coding RNAs have shown to play important roles in plant for controlling flowering time by regulating the expression of key players in flowering time. Among numerous miRNAs, several miRNA families have been confirmed to play important roles in controlling flowering, serving either to inhibit or to promote reproduction. The main players are the *miR156*, *miR159* and *miR172* families. In addition, the *miR319*, *miR390* and *miR399* families also play a role in the control of flowering time [8,9,10,11].

Overexpression of *miR156* reduces the level of target *SPL* genes and causes a late-flowering phenotype [12,13]. A recent study also found that the *miRNA156-SPL3* module regulates ambient temperature-responsive flowering via *FT* in Arabidopsis [14]. When *miR159* was overexpressed, plants flowering time was delayed in SD condition with decreased levels of *MYB33* and *LFY* in Arabidopsis [15]. *miR172* is one of the earliest microRNAs isolated by small RNA sequencing in Arabidopsis [16] and later found in ferns, gymnosperms and the flowering plants, but not in lycopods and moss [17,18]. In Arabidopsis, *miR172* serves as a negative regulator of *AP2* to specify floral organ identity and also acts as a repressor of the AP2-like genes, the *Target of EAT 1* (*TOE1*) and *SCHLAFMU TZE* (*SMZ*) to promote early flowering [19,20,21]. A progressive increase of *miR172* level promotes the juvenile-to-adult transition in maize [3]. Over-expression of *miR172* causes the loss of spikelet determinacy and floral organ abnormalities in rice [22]. In soybean, the overexpression of *miR172c* increases soybean nodule numbers, whereas diminishes endogenous activity of *miR172c*, resulting in reduced nodulation [23]. However, the roles of soybean *miR172* and its AP2-like targets on flowering time are currently unclear.

We also investigated a novel physiological function of *gma-miR172a* in soybean. The analyses of the expression pattern and 5′RACE showed that *Glyma03g33470* was a target gene of *gma-miR172a*, and the ectopic overexpression of *gma-miR172a* in Arabidopsis (Col-0) accelerated flowering both in long day and short day conditions. The results of qRT-PCR analysis indicated that the overexpression of *gma-miR172a* altered the transcriptional profiles of the genes that were involved in flowering control. In addition, *toe1* mutant plants of Arabidopsis could restore its earlier flowering phenotype partially by the expression of *Glyma03g33470*.

## 2. Results

### 2.1. Identification and Analysis of gma-miR172a and Glyma03g33470 Sequences

**Figure 1:**
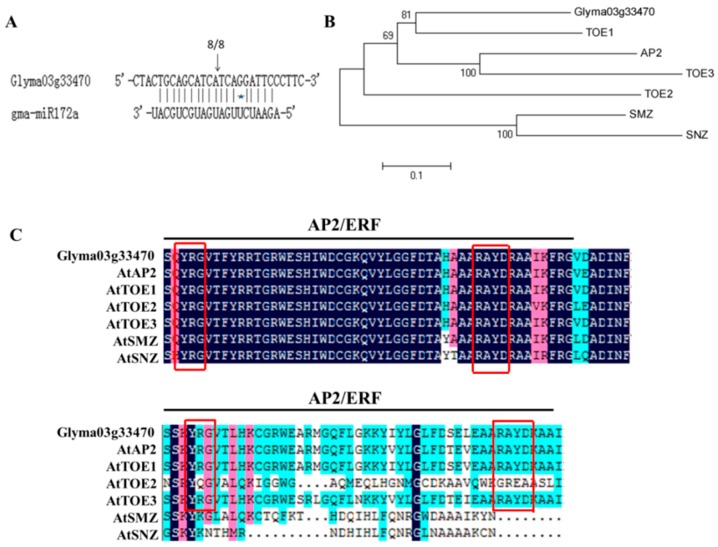
Identification and analysis of *gma-miR172a* and *Glyma03g33470* sequences. (**A**) Cleavage sites of *Glyma03g33470* mediated by *gma-miR172*. The *miR172*-mediated cleavage sites were identified by 5′RACE in the predicted targets. Arrow indicated the cleavage site. The number on the top of sequence alignment represented the frequency of clones corresponding to cleavage site; (**B**) The *Glyma03g33470* homologs of soybean among AP2-Like genes in Arabidopsis were shown by phylogenetic analysis. Phylogenetic tree was constructed using the software MEGA 5.0 through the method of neighbor joining; (**C**) Multiple alignments of the amino acid sequences encoded by *Glyma03g33470* and known AP2-Like genes in Arabidopsis. The two AP2/ERF domains were indicated by the line on the top of sequences. Conserved YRG and RAYD sequence motifs in AP2 domain were denoted by the red frame. Blue, homology 100%; pink, homology >=75%; light blue, homology >=50%.

The *miR172* family of soybean (*gma-miR172*) was encoded by twelve genomic loci (*gma-miR172a* to *gma-miR172l*) based on miRBase version 21.0 (Available at: http://www.mirbase.org) and the precursors of the different members varied (Supplementary Materials Figure S1), but the mature sequences were highly similar. According to the difference of mature sequence, the members of *gma-miR172* were divided into eight categories (Table 1). Eight potential AP2-Like target genes of soybean *gma-miR172* were obtained from PMRD database (Available at: http://bioinformatics.cau.edu.cn/PMRD/) (Supplementary Materials Table S1). 5′ rapid amplification of the cDNA ends (5′RACE) was used to determine whether these putative targets were cleaved by *gma-miR172* with RNA isolated from 20 DAE (Day after emergence) leaves. Among these target genes, PCR bands with the distinct and expected sizes were observed for the *Glyma03g33470* (Supplementary Materials Figure S2). Cloning and DNA sequencing of this amplified product with the 5′ end of the cleavage products to the designated position, the degradation segment of *Glyma03g33470* was obtained and the cleavage sites were between the 10th and 11th nucleotides complementary to *gma-miR172a* (Figure 1A), similar to the other species [19,22,24]. The results indicated that *Glyma03g33470* was the target gene of *miR172* in soybean.

The cDNA sequence of the predicted target gene *Glyma03g33470* was 2238 bp with an open reading frame of 1380 bp and was predicted to encode 459 amino acids, with a predicted molecular mass of 50.27 kDa and a predicted pI (isoelectric point) of 6.27. Phylogenic analysis of the protein sequences revealed that *Glyma03g33470* shared high amino acid sequence identity with *TOE1* of Arabidopsis (Figure 1B). The putative target gene of soybean *miR172*, *Glyma03g33470*, shared high peptide identities with *AP2* and *TOE* genes and contained two AP2 domains, similar to the previous reports on *TOE1* in Arabidopsis [25]. Each AP2 domain possessed two completely conserved sequence motifs: YRG motif and RAYD motif (Figure 1C).

### 2.2. Temporal and Spatial Expression Patterns of gma-miR172 and Their Target Genes in Soybean

**Figure 2:**
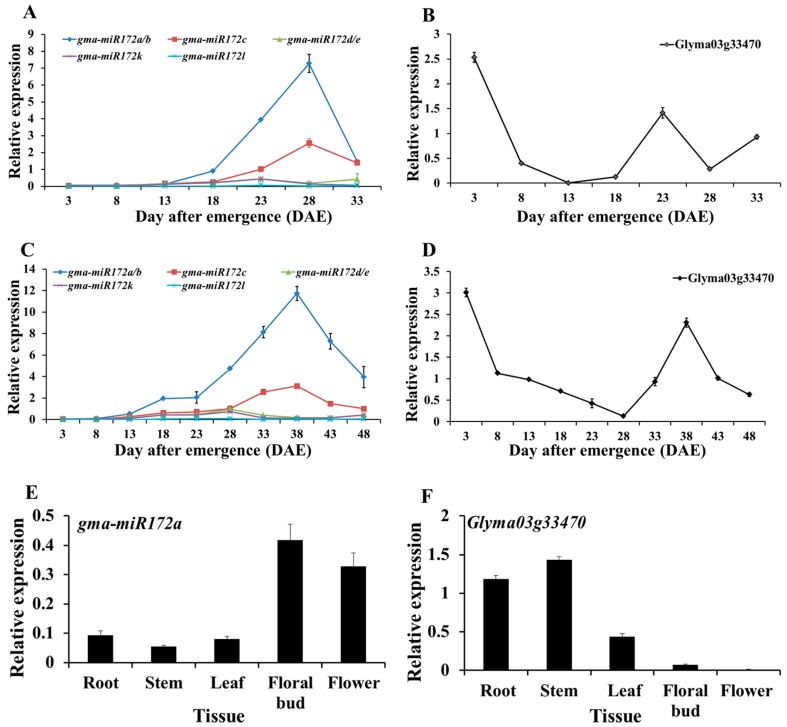
The relative expression levels of *gma-miR172* and *Glyma03g33470* in soybean. (**A**–**D**) Expression levels of *gma-miR172* and *Glyma03g33470* during the development of soybean under SD (short day) (**A**,**B**) and LD (long day) (**C**,**D**) conditions. The leaf samples for RNA extraction were harvested every five days during 3–33 DAE under SD and 3–48 DAE under LD conditions; (**E**,**F**) Tissue-specific expression of *gma-miR172a* (**E**) and *Glyma03g33470* (**F**) under SD conditions. Relative mRNA abundance was determined by qRT-PCR according to method of 2^−ΔΔ*C*t^ based on *C*_t_ values and the *Actin4* gene was used as an endogenous control. All relative expression levels are compared to *Actin* = 1. Data shown were the mean of three independent repeated experiments ± standard deviation.

To determine how the *gma-miR172* was expressed during soybean development, the expression levels of mature sequences of *gma-miR172a/b*, *gma-miR172c*, *gma-miR172d/e*, *gma-miR172f*, *gma-miR172g*, *gma-miR172/h/i/j*, *gma-miR172k* and *gma-miR172l* were analyzed by qRT-PCR. The results showed that *gma-miR172f*, *gma-miR172g* and *gma-miR172h/i/j* could not be detected in young seedlings and all the other five *gma-miR172* members could be expressed in a low level at initial stage, *gma-miR172d/e* and *gma-miR172k* increased slightly subsequently, but the RNA abundance of *gma-miR172a/b* and *gma-miR172c* increased rapidly throughout the entire vegetative phase and peaked at 28 DAE (a flower bud appeared approximately on the 30th day) in the initiation of flowering phase, then gradually decreased during the reproductive phase under SDs (Figure 2A). Interestingly, the abundance of *gma-miR172a* was apparently higher than the other four members during the lifecycle (Figure 2A). Therefore, it was inferred that *gma-miR172a* might play a more important role in flowering time control than the other members. The expression level of *Glyma03g33470* was also analyzed by qRT-PCR using the primer pairs spanning the *miR172* cleavage sites and its mRNA abundance also exhibited a temporal specificity expression pattern during the growth and development stage of soybean. The expression level was quite high in young seedlings, gradually declined as the plants grew and was lowest at 13 DAE. Then, the transcript level became elevated during the flowering phase and declined again during the late reproductive phase under SD (Figure 2B). Under LD (long day) conditions, the expression patterns of *gma-miR172a* and *Glyma03g33470* were similar to those under SD (short day) conditions and *gma-miR172a* with the highest accumulation at the initiation of flowering phase (Flower bud appeared in 42th day approximately) (Figure 2C,D).

To obtain further insights into how *gma-miR172a* regulates *Glyma03g33470*, the transcript levels of mature *gma-miR172a* and *Glyma03g33470* were compared in plant tissues. The expression of *gma-miR172a* was lower in stems, whereas, its expression was much higher in floral buds (Figure 2E). In contrast to *gma-miR172a*, the transcript abundance of *Glyma03g33470* was lower in floral buds and higher in stems (Figure 2F).

### 2.3. Diurnal Rhythm of gma-miR172a and Glyma03g33470

**Figure 3:**
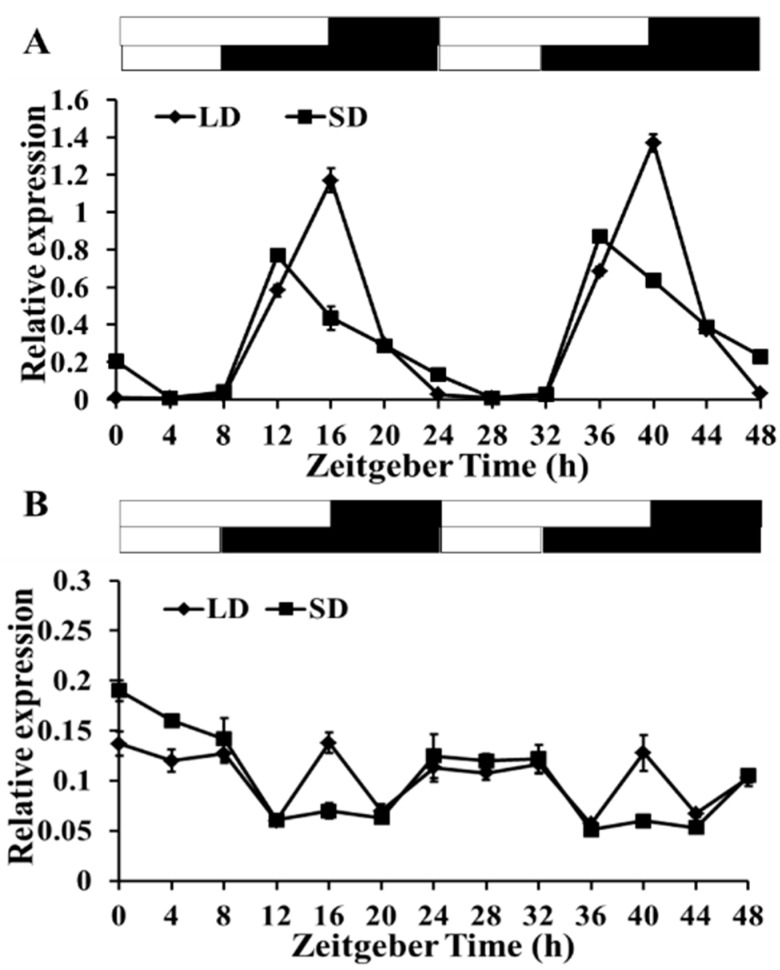
The diurnal rhythm of *gma-miR172a* and *Glyma03g33470* gene expression. (**A**) *gma-miR172a* diurnal expression under SDs and LDs; (**B**) Expression pattern of *Glyma03g33470* under SDs and LDs. The leaf samples for RNA extraction were collected every 4 h during a total of 48 h. White bars represented light and black bars represented dark phases. Relative mRNA abundance was determined by qRT-PCR and the *Actin4* gene was used as an endogenous control. Data shown were the mean of three independent repeated experiments ± standard deviation.

In order to know if the expressions of *gma-miR172a* and *Glyma03g33470* have diurnal rhythm, trifoliate leaves (20 DAE) were sampled every 4 h. The expression level of *gma-miR172a* was higher under LD conditions than SD conditions, as well as *Glyma03g33470* (Figure 3A,B). *gma-miR172a* exhibited a diurnal rhythm both under SD and LD conditions. The gene expression reached a peak at 16 h after dawn under SD and the expression peak was postponed for about 4 h under LD conditions (Figure 3A). The expression level of *Glyma03g33470* decreased gradually after dawn and reached the minimum at 12 h, then increased slightly under LD, but the relative levels were constantly low under SD (Figure 3B), suggesting that the expression of *Glyma03g33470* did not follow the diurnal rhythm pattern.

### 2.4. Over-Expression of gma-miR172a Results in Earlier Flowering in Transgenic Arabidopsis

**Figure 4:**
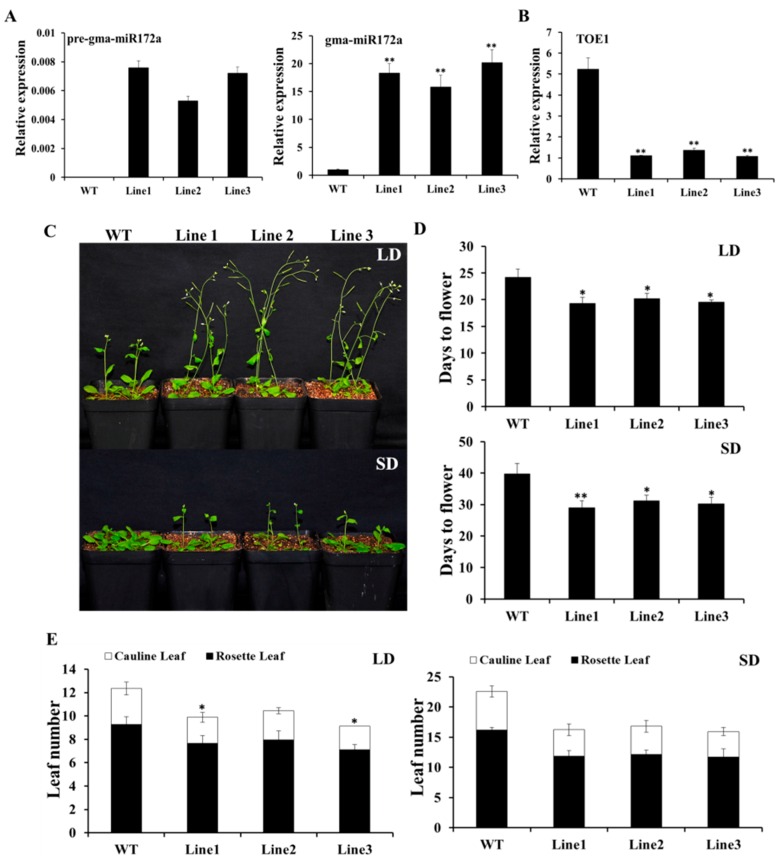
*gma-miR172a* conferred early flowering in transgenic Arabidopsis (**A**,**B**) Relative expression levels of the mature, precursor *gma-miR172a* and *AtTOE1* in *gma-miR172a*-transgenic plant lines were determined by qRT-PCR. *Actin8* gene was used as an endogenous control; (**C**) Flowering phenotype of *gma-miR172a* plants under both LD and SD conditions. Photographs were taken of plants after growing in soil for four weeks; (**D**) Days to flowering of WT and *gma-miR172a* transgenic plants under both LD and SD conditions; (**E**) The average number of total leaves in *gma-miR172a*-transgenic and WT plants at bolting time under both LD and SD conditions. Number of leaves from 20 individual plants at bolting was used for counting and averaging. Significant difference among WT and three *gma-miR172a*-transgenic lines were indicated by asterisks (* *p* < 0.05; ** *p* < 0.01).

In order to verify the function of *gma-miR172a* in the flowering time control, the *gma-miR172a* precursor was genetically transformed into *Arabidopsis thaliana* under the regulation of the cauliflower mosaic virus (CaMV) 35S promoter. The precursor and mature sequences of *gma-miR172a* were all increased in transgenic lines compared to wild type (Figure 4A). *TOE1* was also down-regulated (Figure 4B). The flowering phenotype of the plants both under LDs and SDs were examined by counting the numbers of the total leaves at bolting and the days from germination to bolting. As showed in Figure 4C–E, the *gma-miR172a*-transgenic lines had 9–10 leaves and spent an average of 19–20 vegetative growth days to flowering under LDs, rather than WT plants that had 12 leaves and spent 24 growth days to the flowering time. Under SD, the *gma-miR172a* lines had 16–17 leaves and spent an average of 29–31 vegetative growth days to the flowering time, whereas WT plants had 23 leaves and spent an average of 40 vegetative growth days to the flowering time (Figure 4C–E). The results demonstrated that the over-expression of *gma-miR172a* resulted in earlier flowering in transgenic Arabidopsis.

### 2.5. Up-Regulation of FT, AP1 and LFY in gma-miR172a-Transgenic Plants

**Figure 5:**
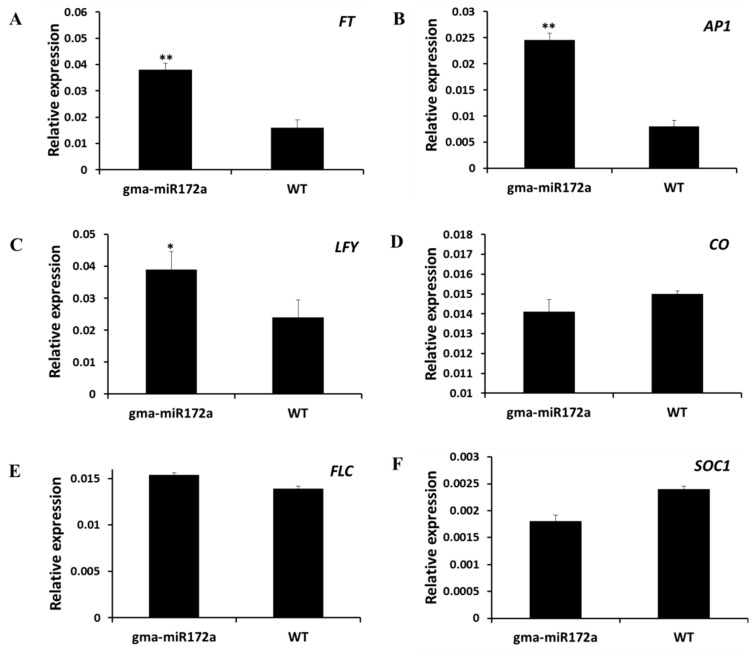
Relative mRNA abundance of flowering-time related genes (**A**) *FT*; (**B**) *AP1*; (**C**) *LFY*; (**D**) *CO*; (**E**) *FLC*; (**F**) *SOC1* in *gma-miR172a* transgenic and WT plants by qRT-PCR. The leaf samples for total RNA extraction were harvested at ZT (Zeitgeber time) 4 from 10-day-old plants grown under LD conditions. The *Actin8* gene was used as an endogenous control. Data shown were the mean of three independent repeated experiments ± standard deviation, a significant difference between *gma-miR172a* transgenic and WT plants was indicated by asterisks (* *p* < 0.05; ** *p* < 0.01).

As *gma-miR172a*-transgenic plants showed an earlier flowering phenotype, the expression levels of flowering time-related genes (including *FLC*, *CO*, *SOC1*, *FT*, *LFY* and *AP1*) in *gma-miR172a*-transgenic plants were further investigated to determine how *gma-miR172a* affects flowering time. The results showed that *FT* and floral homeotic genes, such as *AP1* and *LFY*, obviously increased in the transgenic plants compared to WT plants. The expression of *CO*, *FLC* and the other genes functioning in the autonomous pathway was unaltered compared to WT (Figure 5), indicating that *gma-miR172a* exerted its role by inducing *FT* through a genetic pathway, rather than the autonomous pathway.

### 2.6. toe1 Mutant Plants Restores Earlier Flowering Phenotype by the Expression of Glyma03g33470

**Figure 6:**
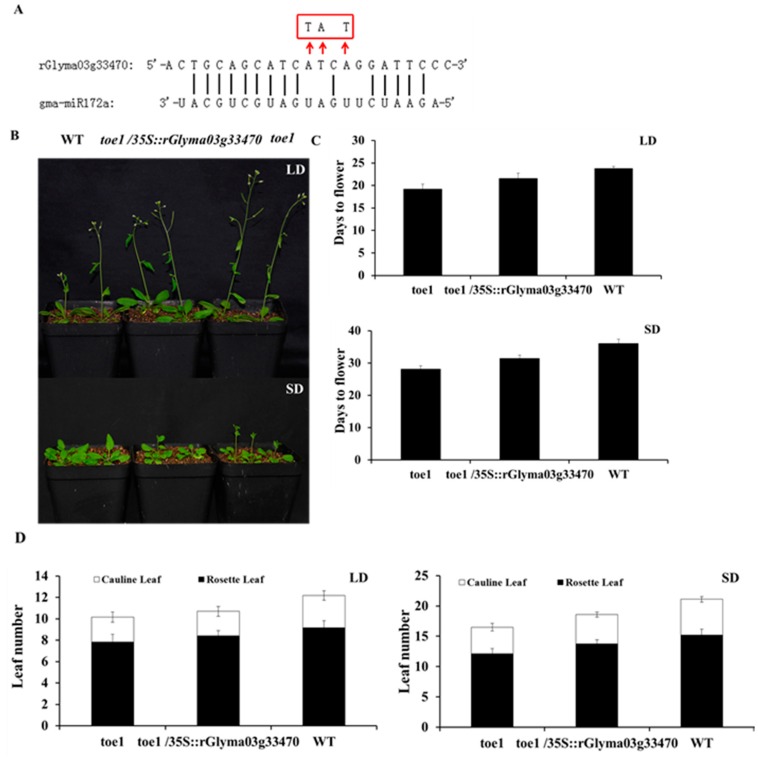
*toe1* plants restores its earlier flowering phenotype by the expression of *Glyma03g33470* under both LD and SD conditions. (**A**) *rGlyma03g33470* rendering the mRNA resistant to *gma-miR172a*-directed cleavage. Red frame indicated the replaced nucleotide; (**B**) Flowering phenotype of *toe1*, *toe1/35S::rGlyma03g33470* and WT plants. Photographs were taken of plants after growing in soil for four weeks; (**C**) Days to flowering of *toe1*, *toe1/35S::rGlyma03g33470* and WT plants; (**D**) Average total leaf numbers of toe1, toe1/35S::r*Glyma03g33470* and WT plants at the bolting both under LD and SD conditions. Number of total leaves from 20 individual plants at bolting was used for counting and averaging.

It has been shown that Arabidopsis *toe1* mutant exhibits early flowering phenotype compared to WT plant [19,25]. To further investigate whether the regulation of *Glyma03g33470* by *gma-miR172a* plays a role in controlling flowering, a version of *Glyma03g33470* mRNA (*rGlyma03g33470*) that carried silent mutations in the *gma-miR172* complementary sites was introduced into *toe1* mutant plants to render the mRNA resistant to *miR172*-directed cleavage (Figure 6A). This complemented line was called *toe1/35S::rGlyma03g33470*.

As shown in Figure 6B,C, the *toe1/35S::rGlyma03g33470* plants flowered a little earlier compared with the WT plant, but still later than *toe1* mutant. Moreover, rosette leaf number and total leaf number of *toe1/35S::rGlyma03g33470* plants were between WT and *toe1* mutant plant (Figure 6D). These data suggested that *toe1* plants could restored its earlier flowering phenotype partially through the expression of *rGlyma03g33470*.

### 2.7. GmGIa Regulates gma-miR172a through miRNA Metabolism in Soybean

**Figure 7:**
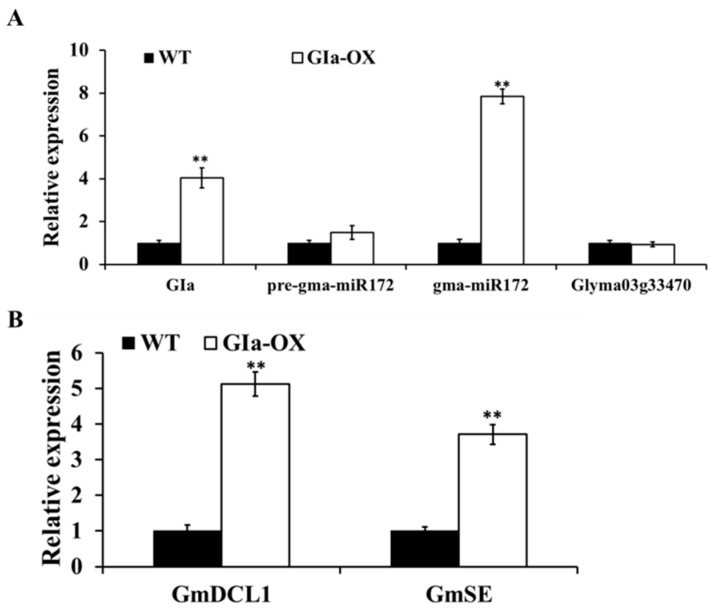
Regulation of *gma-miR172a* and *Glyma03g33470* by *GmGIa*. (**A**) qR-TPCR analysis of *Glyma03g33470*, the precursor, and mature sequences of *gma-miR172a* expression levels in *GmGIa*-ox and WT soybean leaves; (**B**) qRT-PCR analysis of *GmDCL1* (*Glyma19g45060*), *GmSE* (*Glyma04g15990*) expression levels in *GmGIa*-ox and WT soybean leaves. The *Actin4* gene was used as an endogenous control. Data shown were the mean of three independent repeated experiments ± standard deviation, a significant difference compared to the corresponding controls was indicated by asterisks (** *p* < 0.01).

It has been shown that *GI* regulates *miR172* processing rather than the transcription of the *miR172* in Arabidopsis [25]. *GmGIa* is the classical maturity locus E2, which has multiple functions involved in the circadian clock and flowering [26,27]. In order to determine whether *GI* affects the expression of *gma-miR172a* and *Glyma03g33470*, the expression levels of *Glyma03g33470*, precursor and mature sequences of *gma-miR172a* in *GmGIa*-overexpression (*GmGIa*-ox) and WT soybean were examined under LD conditions. Our results showed that there was no difference in the expression levels of *pre-gma-miR172a* and *Glyma03g33470* between the *GmGIa*-ox and WT. However, the mature sequence of *gma-miR172a* was up-regulated by *GmGIa* (Figure 7A).

*Dicer-like1* (*DCL1*) and *SERRATE* (*SE*) are key genes that encoding miRNA processing enzymes [28]. In the present study, the amino acid sequence of Arabidopsis *AtDCL1* and *AtSE* was used as a query in the Phytozome database (Available at: http://www.phytozome.net) to search for homologs in the soybean genome and identified *Glyma19g45060*, *Glyma04g15990* as *GmDCL1* and *GmSE* gene respectively. The expression levels of *GmDCL1* and *GmSE* in GmGIa-ox and WT soybean were detected. As expected, their expression levels were obviously increased in the *GmGIa*-ox soybean (Figure 7B). In conclusion, it is likely that *GmGI* also regulates *miR172* maturation (processing) in soybean rather than *miR172* transcription.

## 3. Discussion

Recently, many studies have revealed that miRNAs plays a crucial role in gene expression regulation and is involved in flowering time control [29,30]. With the development of high-throughput sequencing (next-generation sequencing, NGS) technology, a large number of miRNAs related to flowering and flower development have been identified and studied in several important species, such as trifoliate orange [31], rice [32], hickory [33], xanthoceras sorbifolia [34], poplar [35], and radish [36].

Soybean is an important grain and economic crop around the world. As a typical short-day plant, it has been studied widely by many researchers. Appropriate flowering time is a key factor in order to gain higher yields in soybean. However, the molecular regulation mechanism of the flowering response in soybean remains unclear. In our previous studies, we found that the expression levels of some members of *gma-miR172* could be induced by a shorter day. *gma-miR172* together with its targets are likely regulated by day length in soybean and play an important role in flowering time control [37].

There are 12 members (*miR172a–l*) in the soybean *miR172* family and they are predicted to target some important AP2-like genes, as shown in Table S1. In our study, we found that the relative expression levels of *gma-miR172a/b*, *gma-miR172c*, *gma-miR172d/e* and *gma-miR172k* in soybean are increased as plants grow up to the flowering stage but the expression levels of other members couldn’t be detected in soybean due to the significant differences between the mature sequences. Interestingly, the abundance of *gma-miR172a* was apparently higher than the other four members during the lifecycle. Furthermore, the 5′RACE assays indicated that *miR172a* directed *Glyma03g33470* mRNA cleavage in soybean and *Glyma03g33470* also showed differential expression patterns in different tissues and developmental stages.

*miR172* was confirmed to participate in the flowering control pathway in many plants but we did not find that *miR172* had a diurnal rhythm expression pattern in Arabidopsis [25]. However, our study showed that the expression level of *gma-miR172a* could be affected by day length and had a diurnal rhythm expression pattern under LD or SD conditions (Figure 3). In addition, Zhao found that *GI* didn’t affect the expression level of *GmTOE4a* in soybean [24], but it could affect the relative level of mature miR172 in Arabidopsis [25]. In our study, the expression levels of *Glyma03g3347* and the precursor, mature sequences of *gma-miR172a* in *GmGIa*-overexpression (*GmGIa*-ox) soybean were examined under LD conditions. We found that although no significant difference in the mRNA abundance of *pre-gma-miR172a* and *Glyma03g33470* was detected between the GmGIa-ox and WT, the mature sequence of *gma-miR172a* was up-regulated by *GmGIa*. *DCL1* and *SE* are the key enzymes in the miRNA synthesis pathway. Therefore, the expression levels of homologs for *DCL1* and *SE* in soybean were compared in *GmGIa*-ox and WT. The results demonstrated that the expression levels obviously increased in the *GmGIa*-ox soybean, implying that *gma-miR172a* was affected by *GmGI* through miRNA metabolism in soybean (Figure 7).

In order to further characterize the function of *gma-miR172a*, *gma-mIR172a* transgenic Arabidopsis was generated. *gma-mIR172a* conferred early flowering phenotype in transgenic plants, and decreased the total leaf number compared to WT plants. In addition, the relative mRNA abundance of flowering-related genes (*FT*, *LFY* and *AP1*) was increased in *gma-miR172a* transgenic plants. The expression of *Glyma03g33470* in *toe1* mutant led to the complementation of the early flowering mutation. *miR172* played a negative regulation of *AP2*. It could specify floral organ identity in Arabidopsis [19,38] but in the present study, no difference in floral organ between transgenic Arabidopsis and WT plants was found, inferring that there may be a different regulation mode between Arabidopsis and soybean.

**Figure 8:**
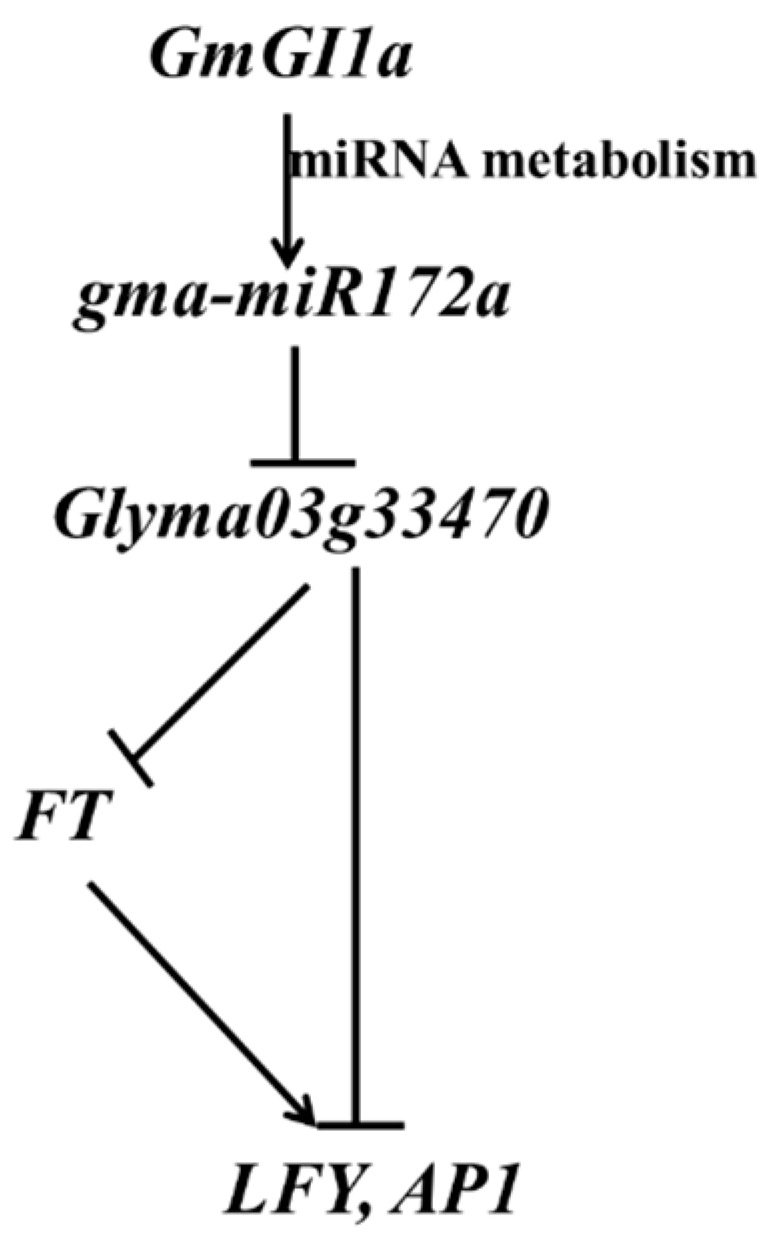
Proposed regulatory pathway of *gma-miR172a* involved in the flowering time control. Arrows represented the up-regulation expression of the gene; T-shaped represented the down-regulation expression of the gene.

Together, the present results demonstrated that a molecular regulation mechanism of *gma-miR172a* and *Glyma03g3347*, played critical roles in the pathway of *GmGIa*-mediated flowering time control in soybean (Figure 8). *GmGI* promotes *miR172* metabolism and represses *Glyma03g3347*. Furthermore, the relative mRNA, abundance of *FT*, *LFY* and *AP1* were also significantly increased due to the over-expression of *gma-miR172a*, conferring the early flowering phenotype in transgenic lines.

## 4. Materials and Methods

### 4.1. Plant Materials and Growth Conditions

In this study, *Arabidopsis thaliana* (Col-0) was used for wild-type control plants and genetic transformation. Seeds of the *toe1* mutant (SALK_069677c) were obtained from the Arabidopsis Biological Resource Center (ABRC, Columbus, OH, USA). Soybean Research Institute of Northeast Agricultural University (Harbin, China) provided the pure seeds of cultivar “DongNong 42” and *GmGIa*-overexpression (transgenic soybean that over expressing *GmGIa*) soybean.

Seeds of *gma-miR172a*-overexpression, *toe1 mutant*, *toe1* complemented line and WT Arabidopsis were surface sterilized with 10 percent hypochlorite, then placed on MS agar medium and stratified at 4 °C for 72 h before being placed at room temperature (22 °C). Subsequently, the ten-day-old seedlings were transferred into 1:1 vermiculite: turfy-soil, cultured under LDs (16 h/8 h light/dark), or SDs (8 h/16 h light/dark).

For expression pattern analysis of *gma-miR172* and *Glyma03g3347*, the seeds of “DongNong 42” were grown under LDs and SDs in the greenhouse. At the 20th day after emergence, fresh and fully unfolding trifoliate leaves for RNA extraction were sampled from three individual plants. Collection of the leaf samples were started at dawn and sampled every 4 h during a total of 48 h for diurnal rhythm expression pattern analysis. To analyse the time course-dependent expression pattern, the fresh leaves from four individual plants were sampled for RNA extraction at 4 h after dawn both under LDs and SDs and sampled repeatedly every five days from 3 to 33 DAE for SDs and from 3 to 48 DAE for LDs. For the tissue-specific expression analysis, the different tissues, including root, stem, trifoliate leaves, flower bud and flowers from soybean were sampled. Tissue materials in each analysis were harvested and stored at −80 °C for RNA isolation.

### 4.2. Real Time RT-PCR Analyses

Tissue materials were harvested as previously mentioned. TRizol reagent (Tiangen, Beijing, China) was used to extract total RNA from the tissues of soybean and Arabidopsis. A miRcute miRNA first-strand cDNA synthesis kit (Tiangen) was used to perform first-strand cDNA synthesis of *gma-miR172*. QuantScript RT Kit (Tiangen) was used to perform cDNA synthesis of other genes. The corresponding mature miRNA sequence was applied as sense primers and antisense adaptor primers were provided in the SYBR Green PCR Master Mix Reagent (miRcute miRNA qPCR Detection Kit, Tiangen). A Chromo4 Real-Time PCR System (Bio-Rad, Hercules, CA, USA) was used for performing the Real-time quantitative RT-PCR. Relative abundance of mRNA and miRNA were determined by qRT-PCR according to the method of 2^−ΔΔ*C*t^ based on *C*_t_ values [39]. *Actin4* gene was used as an endogenous control for soybean and *Actin8* gene was used for an endogenous control in Arabidopsis. The sense primer for qRT-PCR of mature *gma-miR172a* was 5′-AGAATCTTGATGATGCTGCAT-3′. The primers used in real time RT-PCR analyses, including *Actin4*, *Actin8*, *Glyma03g33470* and flowering control genes were shown in Table 2.

### 4.3. RACE Mapping of miRNA Target Cleavage Sites

PMRD database (Available at: http://bioinformatics.cau.edu.cn/PMRD/) provided the putative target genes of *gma-miR172a*. A modified 5′ RLM-RACE assay was used to validate the internal cleavage site in these putative targets with the Marathon^®^ cDNA Amplification Kit (Clontech, Mountain View, CA, USA). 20 DAE leaves grown under SD conditions were used for total RNA extraction. Then the total RNA was used to synthesize cDNA with a 5′ adaptor. According to the manufacturer’s protocol (Clontech), nested PCR was used to amplify the cDNA samples. For carrying out the initial PCR, AP1 (adaptor primer 1 was provided in the kit) and the gene-specific outer primer (Table 2) was used. AP2 and the gene-specific inner primer (Table 2) were used for carrying out the nested PCR with 1 μL of the initial PCR reaction products. Then final PCR bands with the distinct and expected sizes were gel purified and cloned to a pGM-T vector (Tiangen) and eight positive clones were used for sequencing.

### 4.4. Gene Constructs and Generation of Transgenic Arabidopsis Plants

For constructing the *gma-miR172a*-overexpression vector, a 159 bp precursor sequence of *gma-miR172a* cloned from “DongNong 42” was inserted into the pGM-T cloned vector for sequencing. The construct was then recombined into the binary vector pCAMBIA3301. The recombined vector pCAMBIA*gma-miR172a*-3301 was introduced into the *Agrobacterium tumefaciens* strain EHA105 which was used to transform Arabidopsis using the vacuum infiltration method. *rGlyma03g33470* rendering the mRNA resistant to *gma-miR172a*-directed cleavage was constructed by overlapping PCR and the full cDNA was inserted into the clone vector pGM-T, then recombined into the binary vector pCAMBIA3301. The recombined vector pCAMBIA *rGlyma03g33470*-3301 was introduced into the *Agrobacterium tumefaciens* strain EHA105 which was used to transform Arabidopsis *toe1* mutant using the vacuum infiltration method. Primers for gene constructs and overlapping PCR were listed in Table 2.

The screened transgenic lines with phosphinothricin-resistance were further confirmed by using PCR amplification. PCR results showed that the resistance of T_2_ transgenic seeds to phosphinothricin was 3:1, and there were no character segregation in T_3_ transgenic seeds (Supplementary Materials Figure S3), so T_3_ transgenic seeds were chosen for further study.

### 4.5. Flowering Time Measurements

To measure flowering time, WT (Col-0), *toe1* mutant, *toe1* recover and *gma-miR172a*-overexpression transgenic plants were all surface sterilized with 10 percent hypochlorite, then placed on MS agar medium and stratified at 4 °C for 72 h before being placed at room temperature (22 °C). Ten-day-old seedlings were transferred to 1:1 of vermiculite and turfy-soil, grown under LDs (16 h light) or SDs (8 h light) conditions. Flowering times were measured by the number of days from germination to bolting with at least 20 plants. The total leaves number at bolting was also measured.

### 4.6. Statistical Analysis

All the results shown in this study were the mean of three independent experiments ± standard deviation. The data were subjected to Student’s *t* test analysis using SPSS statistical software 17.0 (SPSS Inc., Chicago, IL, USA).

## Figures and Tables

**Table 1:** Members of *miR172* family in soybean and their mature sequences.

Locus	Mature Sequence
*gma-miR172a/b*	AGAAUCUUGAUGAUGCUGCAU
*gma-miR172c*	GGAAUCUUGAUGAUGCUGCAG
*gma-miR172d/e*	GGAAUCUUGAUGAUGCUGCAGCAG
*gma-miR172f*	AGAAUCUUGAUGAUGCUGCA
*gma-miR172g*	GCAGCACCAUCAAGAUUCAC
*gma-miR172h/i/j*	GCAGCAGCAUCAAGAUUCACA
*gma-miR172k*	UGAAUCUUGAUGAUGCUGCAU
*gma-miR172l*	GGAAUCUUGAUGAUGCUGCAU

**Table 2:** Primers used.

Primer Names	Primer Sequences (5′–3′)
Adaptor primer 1	5′-CCATCCTAATACGACTCACTATAGGGC-3′
Adaptor primer 2	5′-ACTCACTATAGGGCTCGAGCGGC-3′
Outer primer	5′-GTGGCGACTAACTAACGGAAGCAAAG-3′
Inner primer	5′-TCACAACTATGACCCACAA-3′
overlapping PCR-F	5′-GAAGATCTCCCAGCAACGGGAGTGGTATGA-3′
overlapping PCR-Rm	5′-ATCCAGTAGATGCTGCAGTAGAGAAGG-3′
overlapping PCR-Fm	5′-ATCTACTGGATTCCCTTCAACAATAAT-3′
overlapping PCR-R	5′-GGGTAACCGTGGCGACTAACTAACGGAAGCAAAG-3′
*qGmACTIN4-F*	5′-GTGTCAGCCATACTGTCCCCATTT-3′
*qGmACTIN4-R*	5′-GTTTCAAGCTCTTGCTCGTAATCA-3′
*AtACTIN8-F*	5′-CGTCCCTGCCCTTTGTACAC-3′
*AtACTIN8-R*	5′-CGAACACTTCACCGGATCATT-3′
*gma-miR172a-F*	5′-AGATCTGTGAAGTCGTTTATGGCTGAT-3′
*gma-miR172a-R*	5′-GGTAACCTTAACAGTCGTTATTTGCGG-3′
*qGmDCL1-F*	5′-AAATGCGGACCTACCAAA-3′
*qGmDCL1-R*	5′-TCAAAGCGAATAACGACA -3′
*qGmSE-F*	5′-CTCACTGGGTGGTTGGTT-3′
*qGmSE-R*	5′-GATACATCCCTCGGCTCA-3′
*qGlyma03g33470-F*	5′-GCTTCTCCGTAGCATCTGG-3′
*qGlyma03g33470-R*	5′-GTGGAGGAATGTCATGTTTG-3′
*AtFLC-F*	5′-GCTCTTCTCGTCGTCTCC-3′
*AtFLC-R*	5′-GTTCGGTCTTCTTGGCTC-3′
*AtCO-F*	5′-AAGGTGATAAGGATGCCAAGGAG-3′
*AtCO-R*	5′-GGAGCCATATTTGATATTGAACTGA-3′
*AtSOC1-F*	5′-TCAGAACTTGGGCTACTC-3′
*AtSOC1-R*	5′-TTCTCGTCGTCTCCGCCTCC-3′
*AtAP1-F*	5′-TAAGCACATCCGCACTAG-3′
*AtAP1-R*	5′-TTCTTGATACAGACCACCC-3′
*AtFT-F*	5′-TGGTGGAGAAGACCTCAGGAAC-3′
*AtFT-R*	5′-TGCCAAGCTGTCGAAACAATAT-3′
*AtLFY-F*	5′-TGTGAACATCGCTTGTCGTC-3′
*AtLFY-R*	5′-TAATACCGCCAACTAAAGCC-3′

## Supplementary Materials

Supplementary materials can be found at http://www.mdpi.com/1422-0067/17/5/645/s1.
