# Supplementary material for: Over-Expression of GmGIa-Regulated Soybean miR172a Confers Early Flowering in Transgenic Arabidopsis thaliana
Source: Int J Mol Sci. 2016 Apr 29;17(5):645. doi: 10.3390/ijms17050645 (PMC4881471; doi:10.3390/ijms17050645)
Supplement: Supplementary file 1 [file ijms-17-00645-s001.pdf]

Supplementary Materials: Over-Expression of GmGIa-Regulated Soybean *miR172a* Confers Early Flowering in Transgenic *Arabidopsis thaliana*

Tao Wang, Ming-Yang Sun, Xue-Song Wang, Wen-Bin Li and Yong-Guang Li

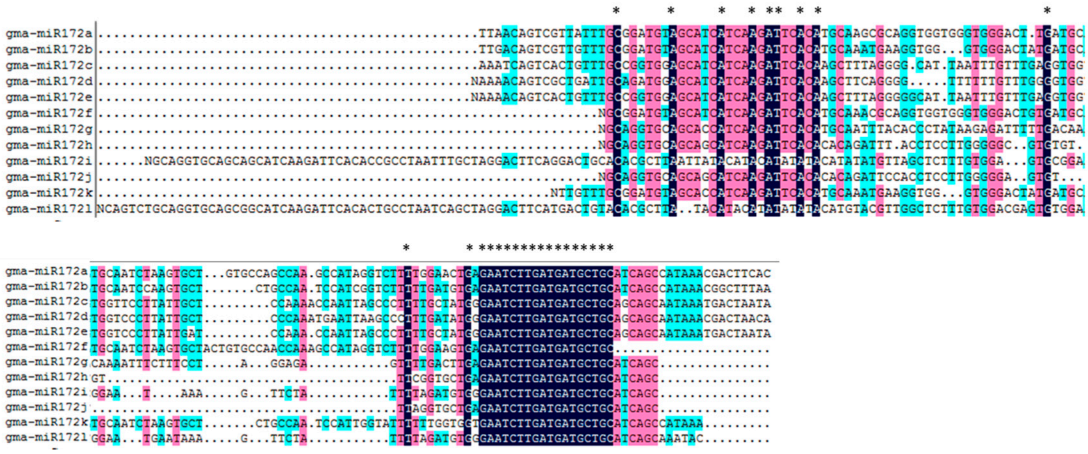

Figure S1. Pre-miRNA sequence alignment of *miR172* family members in soybean. Blue, homology 100%; pink, homology >=75%; light blue, homology >=50%. * represent conserved nucleotides in all pre-miRNAs.

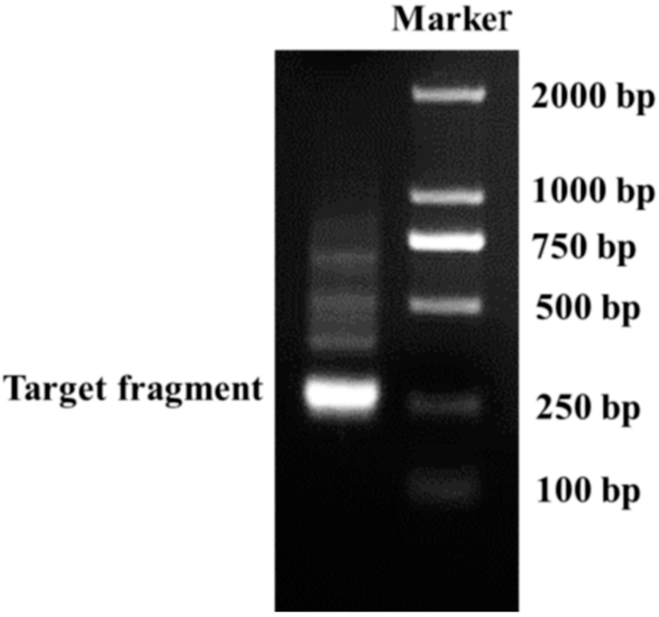

Figure S2. 5′RACE nested PCR product (*Glyma03g33470*), spanning between the primer and cleavage sites, was used for cloning into a vector for DNA sequencing. Lane Marker represents 2000 bp DNA marker.

A

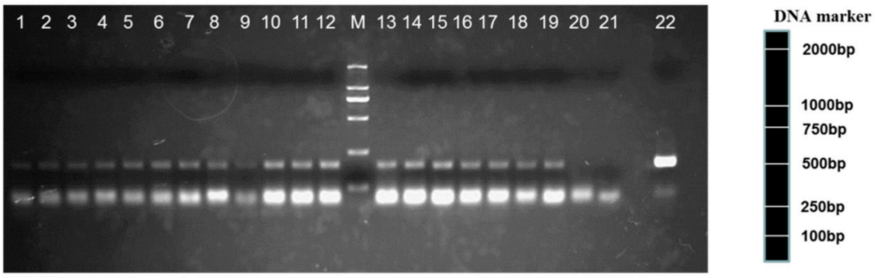

B

Progeny	Line	Number of the Detection	Positive plants	Negative plants	$X^2_{0.05}$	Segregation
T2	5-1	102	66	36	0.333	3:1
	5-2	60	44	16	0	3:1
	5-3	73	56	17	0.333	3:1
T3	Line1	25	25	0		
	Line2	48	48	0		
	Line3	19	19	0		

Figure S3. Identification of transgenic Arabidopsis by PCR amplification. (**A**) Identification of T3 transgenic Arabidopsis by PCR amplification. Lane M: DNA marker; lane 20: Water control; lane 21: Negative control; lane 22: Positive control plasmid; lane 1–19: Transgenic plants. The PCR target fragment is 159 bp; (**B**) Segregation of the *gma-miR172c* in the progeny of self fertilized transgenic Arabidopsis and chi-square test X^2 analysis.

Table S1. Potential AP2-Like target genes of *gma-miR172a* from PMRD (Avaiable at: http://bioinformatics.cau.edu.cn/PMRD/).

Target Gene	Target Description
Glyma17g18640	Q9AXI4 | APETAL2-like protein | Blast E-value: 5E-165
Glyma11g05720	Q9AXI4 | APETAL2-like protein | Blast E-value: 4E-138
Glyma02g09600	B9HLZ7 | AP2 domain-containing transcription factor | Blast E-value: 1E-61
Glyma01g39520	A7PLE5 | Transcription factor APETALA2 (Chromosome chr7 scaffold_20, whole genome shotgun sequence) | Blast E-value: 5E-152
Glyma19g36200	B9SGS1 | Floral homeotic protein APETALA2, putative (EC 1.3.1.74) | Blast E-value: 2E-131
Glyma15g04930	B9SW78 | Protein AINTEGUMENTA, putative (EC 1.3.1.74) | Blast E-value: 8E-133
Glyma03g33470	B9SGS1 | Floral homeotic protein APETALA2, putative (EC 1.3.1.74) | Blast E-value: 4E-129
Glyma05g18170	Q9AXI4 | APETAL2-like protein | Blast E-value: 6E-131
